# When Correction Turns Positive: Processing Corrective Prosody in Dutch

**DOI:** 10.1371/journal.pone.0126299

**Published:** 2015-05-14

**Authors:** Diana V. Dimitrova, Laurie A. Stowe, John C. J. Hoeks

**Affiliations:** 1 Donders Institute for Brain, Cognition and Behaviour, Centre for Cognitive Neuroimaging, Radboud University Nijmegen, Nijmegen, The Netherlands; 2 Centre for Language Studies, Radboud University Nijmegen, Nijmegen, The Netherlands; 3 Center for Language and Cognition Groningen, University of Groningen, Groningen, The Netherlands; 4 Neuroimaging Center, University Medical Center Groningen, Groningen, The Netherlands; The University of Nottingham, UNITED KINGDOM

## Abstract

Current research on spoken language does not provide a consistent picture as to whether prosody, the melody and rhythm of speech, conveys a specific meaning. Perception studies show that English listeners assign meaning to prosodic patterns, and, for instance, associate some accents with contrast, whereas Dutch listeners behave more controversially. In two ERP studies we tested how Dutch listeners process words carrying two types of accents, which either provided new information (new information accents) or corrected information (corrective accents), both in single sentences (experiment 1) and after corrective and new information questions (experiment 2). In both experiments corrective accents elicited a sustained positivity as compared to new information accents, which started earlier in context than in single sentences. The positivity was not modulated by the nature of the preceding question, suggesting that the underlying neural mechanism likely reflects the construction of an interpretation to the accented word, either by identifying an alternative in context or by inferring it when no context is present. Our experimental results provide strong evidence for inferential processes related to prosodic contours in Dutch.

## Introduction

In communication, speech partners exchange information to achieve a level of shared knowledge and mutual beliefs. To facilitate the comprehension process, speakers highlight relevant information in their speech in various ways: in some languages this is achieved by using pitch accent. For example, when answering a question like *Who bought the flowers*?, Dutch speakers accent the new information: *TARA bought the flowers* (accented word in capitals). Importantly, speakers also use accents to correct information which is not aligned with their speech partner’s representation of events: *Did you know that Petra bought the flowers*? *I think TARA bought the flowers*. There is considerable debate over the extent to which such ‘corrective’ accents differ from accents which indicate new information more generally. According to one view, a pitch accent can itself have a ‘meaning’ like *contrast* [1]; according to another, acoustic enhancement of pitch accent may imply added importance but is not specifically associated with a particular meaning [2–3]. The goal of the current experiment was to investigate this issue by examining how the brain deals with comprehending two types of pitch accent produced in new information and correction contexts respectively.

The present study focuses on correction, which should be differentiated from contrast [4–6]. According to [4], *corrective focus* refers to the competition of two alternatives for introduction into the common ground. While *contrastive focus* indicates a juxtaposition between the marked item and alternatives in the preceding discourse, *new information focus* is the introduction of an element into the common ground without alternatives being present in the preceding context. To give an example, in the sentence “TARA bought flowers, but PETRA did not”, two alternatives, Tara and Petra, form a contrastive relation, and many other alternatives are plausible extensions to the discourse. In the sentence “TARA bought flowers, not PETRA”, the alternative “Tara” replaces the alternative “Petra”, under the exclusion of all other possible alternatives. Correction and contrast are thus related as they both involve alternatives, however they are non-overlapping. Whereas correction requires the replacement of one element by a new element, contrast can be viewed as the comparison of multiple alternatives, without any of them necessarily being rejected.

Investigation of the distinction between correction and contrast goes beyond the scope of the present paper, but we refer the reader to our previous study [7], in which we showed that contrastive focus receives a different type of accentuation than corrective focus in Dutch. In the present study we will focus on correction, specifically the explicit replacement of one alternative mentioned in the context by another alternative that is new in the discourse. However, we will discuss evidence suggesting that different accents can carry the distinction between contrastive focus vs. new information focus as well as evidence with regard to corrective accent, as it bears on the general issue of whether accents can be directly associated with meanings.

Although we know that some languages differentiate new information focus from contrastive or corrective focus by prosody, this may not be the case for all languages. For instance, English and German employ contrastive accents and new information accents [8–12]. English and German speakers use accents with rising pitch to express contrastive focus, and upon perceiving such contrastive accents (but not new information accents), listeners anticipate contrastive referents in the discourse [12–13]. This suggests that intonation contours can have a meaning. For Dutch, however, this debate has centered around the issue of whether there is in fact a contrastive and/ or corrective accent at all. According to some studies, Dutch does not have a separate *corrective focus accent* that differs phonologically or phonetically from *new information (narrow) focus accent*, although both types of focus accent were distinguishable from *broad focus accents* used in sentences where all information is equally new [14]. In their production study, [14] found that when speakers correct information in dialogue as in *Do you want to stay in Montfort*? *No*, *we want to stay in Manderen*, the corrective information *Manderen* is produced with the same pitch accent as in answers to wh-questions with narrow focus on that element like *Where did you stay*?. However, since in this study corrective answers always started with “no”, which is itself a lexical marker of correction, highlighting corrective information by accent may have no longer seemed necessary to the speaker. If no such lexical cues are present, for instance as in the dialogues like we use in the current experiment: *Did you know that Maria bought the flowers*? *I think TARA bought the flowers*, prosody may play a more important role for signaling correction.

In line with this suggestion, other studies provide evidence that Dutch speakers do disambiguate corrective from non-corrective meanings by the type of pitch accent they use [15–17]. Following questions like *Do you want to go from Eindhoven to Swalmen*?, the corrective reply “no” is produced with a rising pitch accent L*H (low tone (L) in the accented syllable, followed by a rise to a high tone (H); annotations according to the *Tones and Break Indices* system [18], based on the Autosegmental Metrical Theory of [19]). Following requests like *Do you want me to repeat the connection*?, the non-corrective reply “no” is realized with a pitch accent with falling pitch H*L (high pitch followed by a low pitch), which is the default focus accent in Dutch [20]. In the present study, we found this same pitch accent distinction in answers with corrective or new information focus produced by uninstructed speakers, as discussed in more detail in the section *Acoustic analysis* in experiment 1 below. In the following, we will refer to these accents as corrective accent (L*H) and new information accent (H*L) for the ease of the reader. Similar evidence for a dissociation between corrective and new information accents has been found in perception studies on Dutch [16], where listeners interpret L*H accents as corrective and H*L accents as non-corrective. In fact, accents with rising pitch have also been linked to correction [21] and are perceived as more emphatic than the default focus accent with falling pitch [22]. Nonetheless, the Dutch L*H pitch contour is claimed to not carry a corrective meaning *per se* and is not considered part of the phonological system [20].

The goal of the present ERP study is to test the processing consequences of highlighting words by corrective accents and new information accents in Dutch single sentences and in dialogues where alternatives are present or absent, and to investigate the neural mechanism that underlies the processing of corrective prosody. If corrective and new information accents evoke distinct interpretations, we should see a difference between them even in sentences without context. Showing that the two pitch accents in Dutch are processed differently would suggest that listeners are sensitive to their differences. Finding out whether this is indeed the case is the goal of the first experiment.

The second issue addressed here is what role context plays when interpreting the meaning of sentences with corrective and new information accents. This will be investigated by embedding sentences with corrective and new information accents in dialogues where the answer provides either new information or corrects information presupposed in the question. It has been amply demonstrated that listeners are sensitive to the accentuation of new information in context. A previous ERP study on dialogue comprehension [7] showed that Dutch listeners are sensitive to the *placement* of accents in context, similarly to listeners of other Germanic languages [22], [24]. We reported a specific neural mechanism reflecting the processing of accented words in the form of an “accent positivity”. In incongruous dialogues like *Did the club give a bonus or a fine to the player*? *They gave a fine* (= focus) *to the PLAYER* (= background), the superfluous accent on background elicited an N400 effect, but the missing accent on focus did not, which we took as evidence that listeners do not deal similarly with overspecified and underspecified prosodic prominence. We also found that both kinds of incongruous prosody gave rise to late positivities, which we related to the listener’s attempts to integrate the prosodically misaligned information in the larger discourse.

In the current experiment, the goal is to investigate the response to an *overspecified* vs. appropriate corrective accent in and out of context. A modification of the response when the corrective accent is appropriate to the context would provide a strong support for the hypothesis that the corrective accent has its own meaning. In both of these experiments, we might expect either a response to incongruous prosody similar to the negativity found by [7], or a general response to a pragmatic incongruity as suggested by the positivity reported by [25]. However, a positivity in response to accent can have multiple interpretations, as shown by an ERP study on corrective prosody in German dialogues [12]. Participants listened to questions like *Did he promise to support FRAUKE*? and *What did he promise you*?. In the answers *He promised to support ANNA and to clean the kitchen*, *ANNA* carried either a corrective or a new information accent, which have distinct pitch contours in German. The authors found a clear response to accent incongruity in context, a late positivity that was interpreted as a modulation of the CPS component (Closure Positive Shift). The CPS component has previously been linked to processes of prosodic phrasing [26].

As *Anna* occurred at a phrase boundary as well as carrying an incongruous accent, it is difficult to decide whether the late positivity reflected processing of the phrase boundary and/or of accentual incongruity. It is also somewhat problematic that listeners performed a concurrent prosody judgment task on each trial, which may have shifted attention to prosodic congruity. In the present study accented words did not occur at a phrase boundary, nor was a prosodic judgment task used. If we find a late positivity in response to the manipulations of prosody, it must be related to its interpretation rather than directly to prosodic phrasing or task demands, thus allowing us to unambiguously identify the underlying neural mechanism of processing corrective and non-corrective accents in Dutch.

### The present study

The present study aims to show whether sentences with corrective pitch accents engage a specific neural mechanism which is distinguishable from that of sentences with new information pitch accents and if so, whether its timing and nature are affected by the presence and type of discourse context. To avoid interference from a meta-linguistic awareness of prosody we did not use a prosodic judgment task. We employed a semantic relatedness task in 25% of all trials to encourage participants to pay attention to the meaning of the stimuli. We first presented sentences in isolation (experiment 1). With no context and alternatives present, the acoustic difference between corrective and new information accents could be reflected in the stage of early sensory processing. More interestingly, if the two pitch accent patterns lead to differences in interpretation, their processing is more likely to show up as a relatively late neural response.

In experiment 2, sentences were embedded in dialogues that either favored or disfavored a corrective interpretation, in order to test whether the responses to the two accents are affected by the presence of alternatives in the context. Under the hypothesis that the two accent patterns are interpreted differently, we should find clear indications in the ERPs, in response to using corrective accent in new information context and vice versa.

Table 1 displays the experimental conditions. In experiment 1 we presented single sentences that were cut out of two different kinds of dialogue (see Table 1 for an example of the materials). In the new information accent condition, sentences were preceded by wh-questions that requested new information about some referent (e.g., *Who bought the flowers*?). In the corrective accent condition, sentences were preceded by yes/no-questions about a given human referent (e.g., *Did you know that Maria bought the flowers*?*)*. Corrective questions contained a referent *Maria* which was presupposed and served as an alternative to the referent *Tara* in the answer.

**Table 1:** Experimental conditions.

	New information accent	Corrective accent
**New information focus context**		
Wie heeft de bloemen gekocht?	Volgens mij heeft TARA (H*L) de bloemen gekocht.	Volgens mij heeft TARA (L*H) de bloemen gekocht.
*English*: *Who bought the flowers*?	*I think TARA bought the flowers*. *(lit*.: *According to me has Tara de flowers bought*.*)*	
**Corrective focus context**		
Wist jij dat Maria de bloemen heeft gekocht?	Volgens mij heeft TARA (H*L) de bloemen gekocht.	Volgens mij heeft TARA (L*H) de bloemen gekocht.
*English*: *Did you know that Maria bought the flowers*?	*I think TARA bought the flowers*. *(lit*.: *According to me has Tara de flowers bought*.*)*	

Table 1 displays one example of the experimental conditions. In experiment 1 participants listened to only the answer sentence in the two accent conditions, and in experiment 2 participants listened to the full dialogue in all four conditions.

The answering sentences always started with the phrase *volgens mij*, which is approximately translated as “I think” (*I think TARA bought the flowers*). In English *I think* may imply a contrastive interpretation, however, the Dutch the phrase *volgens mij* (literally: *according to me*), does not carry this implication. *Volgens mij* introduces an epistemic uncertainty that allows material in the question to be repeated felicitously in the answer. Without the phrase, the most natural answer to *Who bought the flowers*? would be a single noun such as *Tara*. This answer would not be felicitous in corrective contexts like *Did you know that Maria bought the flowers*?, which require a longer answer, such as *(No*,*) Tara bought the flowers*. We avoided using lexical markers of correction like “no”, as they may affect sentence and prosody processing, as discussed with regard to the results of [14]. In addition, “no” would be infelicitous in simple non-corrective contexts. By using *volgens mij*, the answer sentence sounds natural in both contexts and also precludes issues with prosodic boundaries. *Volgens mij* also leads to inversion of the subject and verb positions in Dutch (*volgens mij*-verb-subject), so that the target *Tara* is not in the vicinity of the sentence initial boundary, which might affect the neural response to prosody [27]. Importantly, the phrase does not itself introduce an adjacent prosodic boundary which could also elicit additional ERP effects such as the CPS [26]. In the absence of a context question, the accent on *Tara* is the only prosodic cue that might induce a corrective or a new information reading of the sentence. Special care was taken to match sentences on length (in words), target word frequency, and syntactic structure by using a within item design. Since semantic and prosodic processing interact [28], we avoided elaborate semantic processing by using targets that were semantically shallow proper names like *Tara*. All target nouns had lexical stress on the initial syllable that always contained a long vowel, in order to avoid variation in accent identification points [29]. These strict stimulus matching procedures help avoid alternative explanations of the results. Prior to the actual experiment we conducted two behavioral pretests to examine whether listeners are sensitive to the congruity of accent and context combinations (pretest 1) and whether they interpret the two pitch accent types differently (pretest 2).

### Behavioral pretests

#### Pretest 1

The goal of pretest 1 was to identify if participants considered the use of corrective pitch accents in a new information context and *vice versa* a mismatch. Seventeen students from the Faculty of Arts who did not participate in the ERP experiments listened to a subset of the experimental dialogues. Their task was to indicate the degree to which question and answer matched, on a scale from 1 (= very bad match) to 7 (= very good match). No definition of ‘match’ was given. To provide anchor points for the rating scale, we added fillers where new information was appropriately accented or where a repeated word was inappropriately accented as new information, as in *Did they give a bonus or a fine to the player*? *They gave a bonus to the PLAYER* [7].

**Fig 1:**
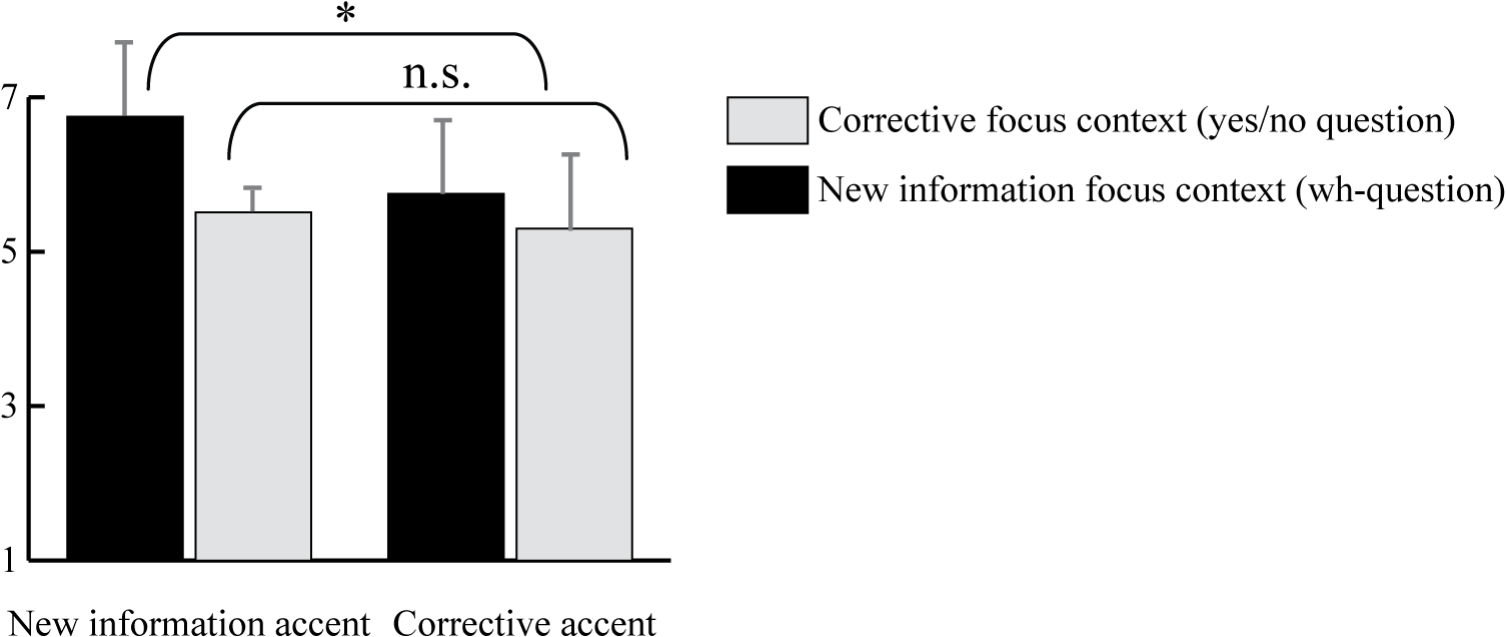
Results of pretest 1. The bars indicate how listeners judged the match between a question and an answer (1 = no match; 7 = match). Accented words carried a corrective accent (L*H) or a new information accent (H*L), which occurred in a new information focus context (wh-question) or in a corrective focus context (yes/no-question).

As Fig 1 shows, all accent-context combinations were judged as relatively good matches on a 7-point Likert scale (all mean values > 4, which is the midpoint of the scale). The results of an ANOVA showed a significant interaction of *Accent* (corrective accent L*H vs. new information accent H*L) and *Context* (yes/no question vs. wh-question) (F_1,16_ = 39.5; p<.001). Follow-up tests revealed that the match between new information accents and new information contexts was judged as significantly higher than the three other conditions, which did not differ significantly. The results of Pretest 1 show that despite their preference for default new information accents, listeners do not perceive new information accents in corrective replies or corrective accents in new information replies as particularly problematic, as long as the content matches the context. Listeners thus judge either prosodic realization as a relatively appropriate answer to both types of question. Though the various combinations of accent and context do not appear to be problematic for listeners, the behavioral rating results do not provide insights into the nature and time course of on-line processing of the two accent types. This was tested in the ERP study.

#### Pretest 2

In pretest 2 we investigated whether participants *interpret* the two pitch accents differently. 15 first-year students from the Faculty of Arts who did not take part in Pretest 1 or in the ERP experiments listened to sentences like *I think TARA bought the flowers* where *Tara* was produced with a corrective or a new information accent. The task was to write down what sentence could have occurred prior to this sentence (for a similar design, see [30]). We hypothesized that the type of reconstructed context will inform us about how accented information is interpreted. We included filler sentences that never started with *I think* and where words carried new information accents in different sentence positions (*They gave a BONUS to the player*; *They gave a bonus to the PLAYER*). Participants wrote down their answers on a blank sheet of paper.

We found substantial individual differences in how sensitive participants appeared to be with respect to prosody. Seven out of fifteen participants always reconstructed a wh-question like *Who bought the flowers*? as a context to the target sentence, irrespective of what type of accent was produced on *Tara*. Six participants reconstructed different contexts based on the type of accent on the proper noun. When *Tara* carried a corrective pitch accent, they reconstructed a statement which contained an alternative to the accented word such as *John bought the flowers*. Four participants sometimes wrote down a corrective context even when *Tara* carried a new information pitch accent. The data show that some listeners indeed interpret corrective accents as implying correction and take the effort to construct a context with an alternative.

### Ethics statement

The treatment of the participants conformed to APA and BPS ethical standards. The experimental protocol was approved by the *Medical Ethical Committee of the University Medical Center Groningen* (METc UMCG). Participants gave informed written consent for participation in accordance with the Declaration of Helsinki.

## Experiment 1

Experiment 1 investigates whether Dutch listeners are sensitive to the type of pitch accent (corrective accent vs. new information accent) in single sentences without a preceding context.

### Methods

#### Participants

Thirty-five right-handed Dutch native speakers (19 female, age 18–29, mean 21) with normal or corrected-to-normal vision participated in the experiment. None of the participants reported any neurological, psychological, language or hearing disabilities; none of them studied Linguistics. The inclusion criterion was set to a minimum of 60% valid data on any electrode used in the analysis in any condition; as a result, four participants were excluded, and data analysis was performed on the data of the remaining 31 participants.

#### Materials

Stimuli were 120 sentences like *Volgens mij heeft Tara de bloemen gekocht* (*I think Tara bought the flowers*). Targets were proper names like *Tara* which do not require elaborate semantic processing. All proper names were matched for frequency on the basis of number of hits each received in the Dutch part of the Google search space. Targets had lexical stress on the initial syllable, which had a long vowel. Target sentences were recorded in short dialogues (Table 1; for the complete list of experimental stimuli see S1 Table). Each sentence was a reply to one of two questions: 1) a yes/no question with an alternative proper name (*Did you know that Maria bought the flowers*?), or 2) a wh-question without a proper name (*Who bought the flowers*?). Sentences were pronounced by two Dutch native speakers who were naive with respect to the research question. The speakers were instructed to distinguish the conditions in their pronunciation and to avoid a pause after *volgens mij*, in line with normal prosodic phrasing in Dutch. We did not instruct the speaker how to accent words in the answer: she could either produce high peaks and prolong the duration of accented words, or she could produce distinct pitch accents in the different conditions. Our speaker chose the latter strategy and produced the target *Tara* with different pitch accents in the corrective focus and in the new information focus conditions. In experiment 1 we removed the questions and participants only listened to the answers of the recorded dialogues. Stimulus pairs were distributed over two lists using a Latin square. In each list half of the stimuli contained a new information accent on the subject (n = 60) and the other half contained a corrective accent on the subject (n = 60).

#### Acoustic analysis

We measured the acoustic duration and fundamental frequency (f0) of targets and the pre-target matrix clause “volgens mij heeft” in PRAAT [31]. As Table 2 shows, overall sentence length did not vary between conditions. In the pre-target clause, conditions differed in pitch and duration: sentences with corrective prosody had a shorter matrix clause and overall lower minimal and maximal frequency than sentences with non-corrective prosody. Pitch range, that is, the difference between maximal and minimal pitch, did not differ between the two conditions. In the target region, the conditions differed only in the pitch domain, with targets with corrective accents showing a higher pitch and a larger pitch slope than targets with non-corrective accents.

**Table 2:** Acoustic stimulus characteristics.

	Pre-target					Target word				
	NA		CA		*p*	NA		CA		*p*
	M^a^	SD^a^	M	SD		M	SD	M	SD	
Duration (ms)	372	39	355	46	*** ^b^	354	53	372	53	***
F0 min (Hz)	172	37	138	22	***	167	26	189	39	***
F0 max (Hz)	224	17	190	18	***	242	24	288	30	***
F0 range (Hz)	52		52		*n*.*s*. ^b^	75		99		***

Acoustic measures were performed for the corrective accent condition (CA) and the new information accent condition (NA). For all target words and the pre-target material (phrase “volgens mij” + auxiliary) we measured duration (in milliseconds; ms), minimal and maximal fundamental frequency (f0) (in Hertz; Hz) and pitch range (difference max f0—min f0).

^a^The values represent mean averages (M) and the corresponding standard deviations (SD).

^b^The symbols indicate significance levels:

*** corresponds to p<.001

n.s. corresponds to p>.05.

**Fig 2:**
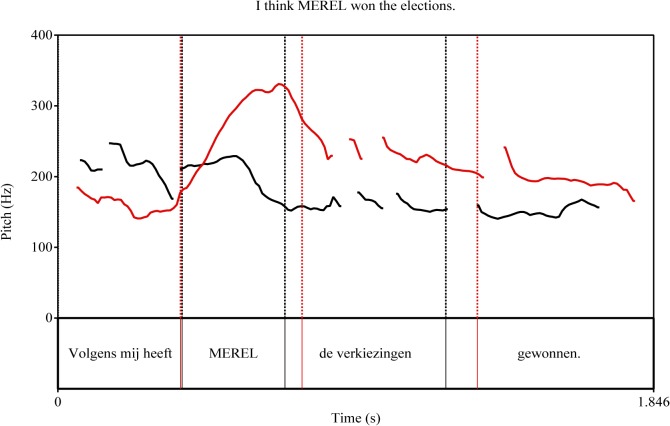
Pitch contours of experimental conditions. The figure shows two examples of the sentence “Volgens mij heeft MEREL de verkiezingen gewonnen” (English: *I think MEREL won the elections*) are displayed: with a new information accent with falling pitch (H*L, black lines) and with a corrective accent with rising pitch (L*H, red lines).

We annotated the pitch contour of each sentence according to the ToDI framework (*Transcription of Dutch Intonation* [20]). The examples of pitch contours are displayed in Fig 2. Accented words in the corrective focus condition were realized with rising pitch accents (L*H) and belonged to a %L L*H L% contour (% for initial or final boundary), referred to as “delayed fall” [20]. As seen for corrective “no”-replies in [15–17], our naive speakers produced accented words in the new information focus condition with falling pitch accents (H*L) in a %L H*L L% contour, called “fall” [20]. No pauses or phrase breaks occurred after *volgens mij* or anywhere in the sentence. Fig 2 indicates that there are prosodic differences on *volgens mij*, which could have been noticeable for the listener prior to the target word. We will return to this issue in the paragraph on ERP Analysis.

### EEG procedure

Participants were seated in an electrically shielded and sound attenuated room and first completed a practice session. To avoid eye movement artifacts, participants fixated on a black cross against a grey screen while they listened to the stimuli via loudspeakers. The fixation cross appeared 100 ms prior to sentence onset and remained on the screen until 1500 ms after the stimulus offset. Participants performed a semantic decision task following 25% of the sentences. A probe word appeared on the screen and they indicated by means of a button press whether it was semantically related to the sentence they had just heard. Related and unrelated probes appeared equally frequently. After the response (or after the 1500 ms silent period), participants were invited to blink (2000 ms).

The EEG was recorded at 250 Hz from 64 Sn channels placed according to the international extended 10–20 system (Electro Cap International) and amplified against the average of all connected inputs of the amplifier (TMS international). The amplifier measured DC without a highpass but with a digital FIR filter (67.5 Hz cutoff) to avoid aliasing. Electrodes were re-referenced offline to the algebraic average of the left and right mastoid electrodes. Vertical eye movements and blinks were monitored via electrodes below and above the left eye, and horizontal movements from electrodes at the left and right canthus of each eye. Impedances were kept below 5Ω.

### ERP analysis

All data were filtered offline with a band-pass filter of 0.01–30 Hz. Trials with movement and ocular artifacts or electrode drifts (± 75 μV voltage maximum) were rejected. The number of rejected trials did not differ across conditions. EEG analysis was performed on 97.5% of the data. Segments of 1300 ms duration were time-locked to the target word onset and at the sentence onset and corrected against a 200 ms pre-stimulus baseline for both. The condition-specific averages were then imported in the Matlab toolbox Fieldtrip [32]. We computed grand averages for each condition, keeping individual trials of every subject, and performed statistical analysis using cluster-based random permutation tests [33]. This approach controls for Type-1 errors that arise due to the multiple comparisons of electrodes and time bins that are common for large data sets in EEG studies. In the random permutation test, a simple dependent t-test is performed on each data sample (i.e., electrode x time pair). If a set of spatially adjacent data samples (i.e., neighbors) exceed the pre-set significance level of 5%, the samples are grouped into clusters. Then a cluster-level test statistic is calculated based on the sum of the *t* statistics within every cluster. In a next step the two conditions of each participant are randomly assigned to one of two sets under the assumption that the two sets do not differ. In this way a null distribution is created and calculated in 5000 randomization steps. Finally, the actually observed cluster-level statistics are compared against the null distribution and all clusters within the lowest and highest 2.5% are considered cases where the null hypothesis can be rejected. That is, if a cluster statistic shows a value of p<.05, the null hypothesis suggesting that the two clusters are the same can be rejected. In experiment 1 we tested for a main effect of *Accent* and compared trials with a corrective accent to trials with a new information accent (CA vs. NA).

Based on previous findings in the literature, we defined three time windows of interest: *100–300 ms* (early P2 window for sensory processing of accent, as reported in [7], [27], [34]), *300–500 ms* (N400 component, which is modulated by accentuation, as shown by [7], [28], and *500–1000 ms* (late window for prosody integration in discourse, cf. [7], [23], [28], [35–36]). We time-locked ERPs to the target onset and computed cluster-based random permutation tests on activity averaged across these time windows. We additionally tested if prosody modulated processing prior to the target word. To this end, we calculated ERPs time-locked to sentence onset and analyzed the time window of 0–400 ms post sentence onset, which corresponds to the baseline period in the target onset analysis. Importantly, time-locking ERPs to sentence onset does not allow us to precisely determine the onset of the target due to jitter related to the auditory presentation of stimuli. Therefore ERPs time-locked to target onset allow for more reliable insights into the processing of target accentuation.

### Results of Experiment 1

#### Behavioral results

Participants judged whether a probe word was semantically related to the preceding sentence in 25% of all trials. The average accuracy was 91%, which suggests that participants paid attention to the stimuli.

#### ERP results time-locked to target onset

**Fig 3:**
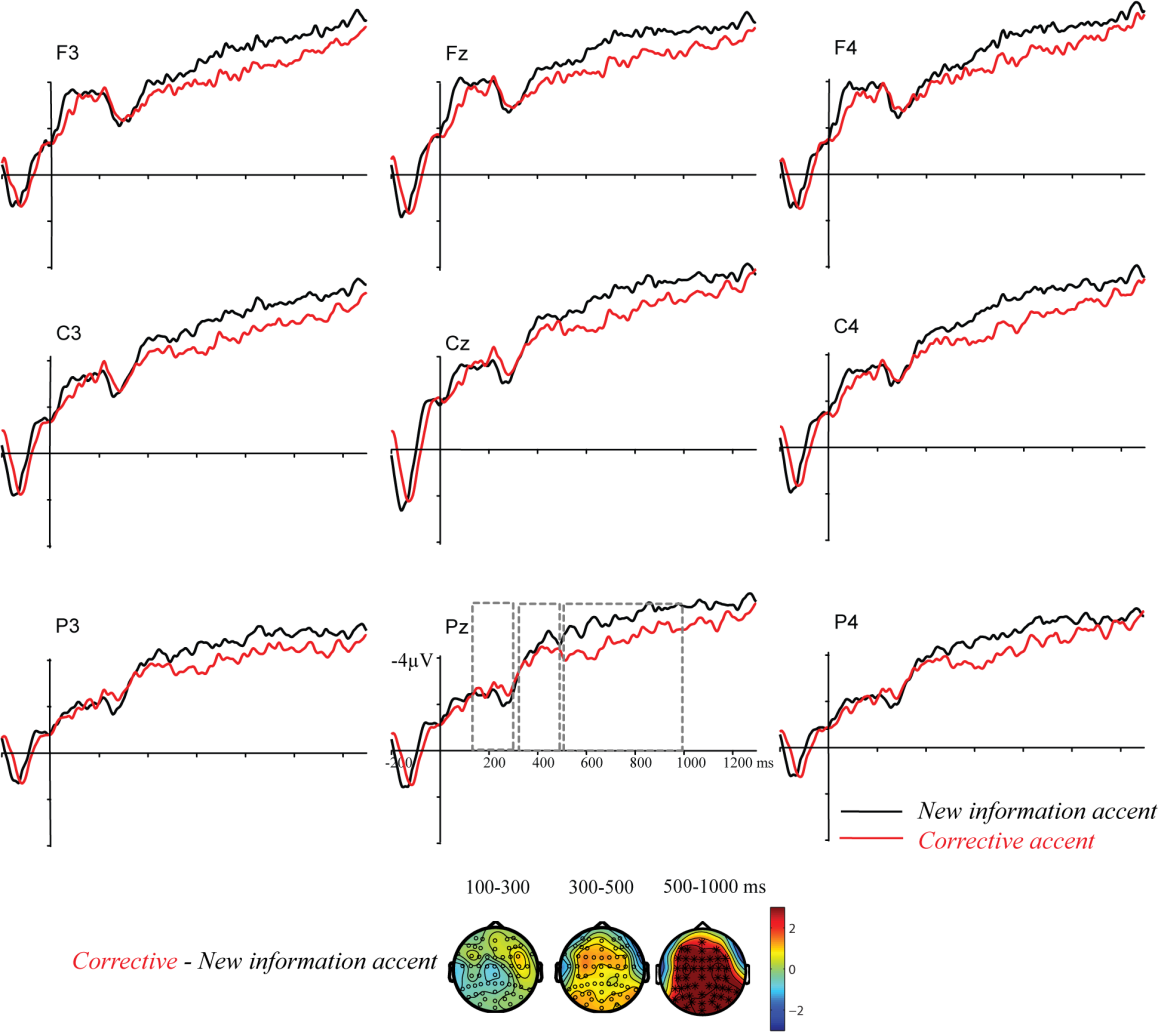
ERPs to single sentences in experiment 1. ERPs are time-locked to the onset of the target *Tara* in the two experimental conditions: (1) corrective accent (red lines) vs. (2) new information accent (black lines).

ERPs time-locked to target onset are displayed in Fig 3.

*100–300 ms*: The cluster-based permutation tests did not reveal any difference between trials with a corrective accent and trials with a new information accent (CA vs. NA; no clusters).

*300–500 ms*: Also in this window there was no difference between targets with a corrective accent vs. a new information accent (CA vs. NA; no clusters).

*500–1000 ms*: The cluster-based permutation test revealed a difference between trials with corrective accents vs. trials with new information accents (CA vs. NA, p<.001). The ERPs show that targets with corrective accents elicited a positive waveform relative to targets with new information accents.

#### ERP results time-locked to sentence onset

**Fig 4:**
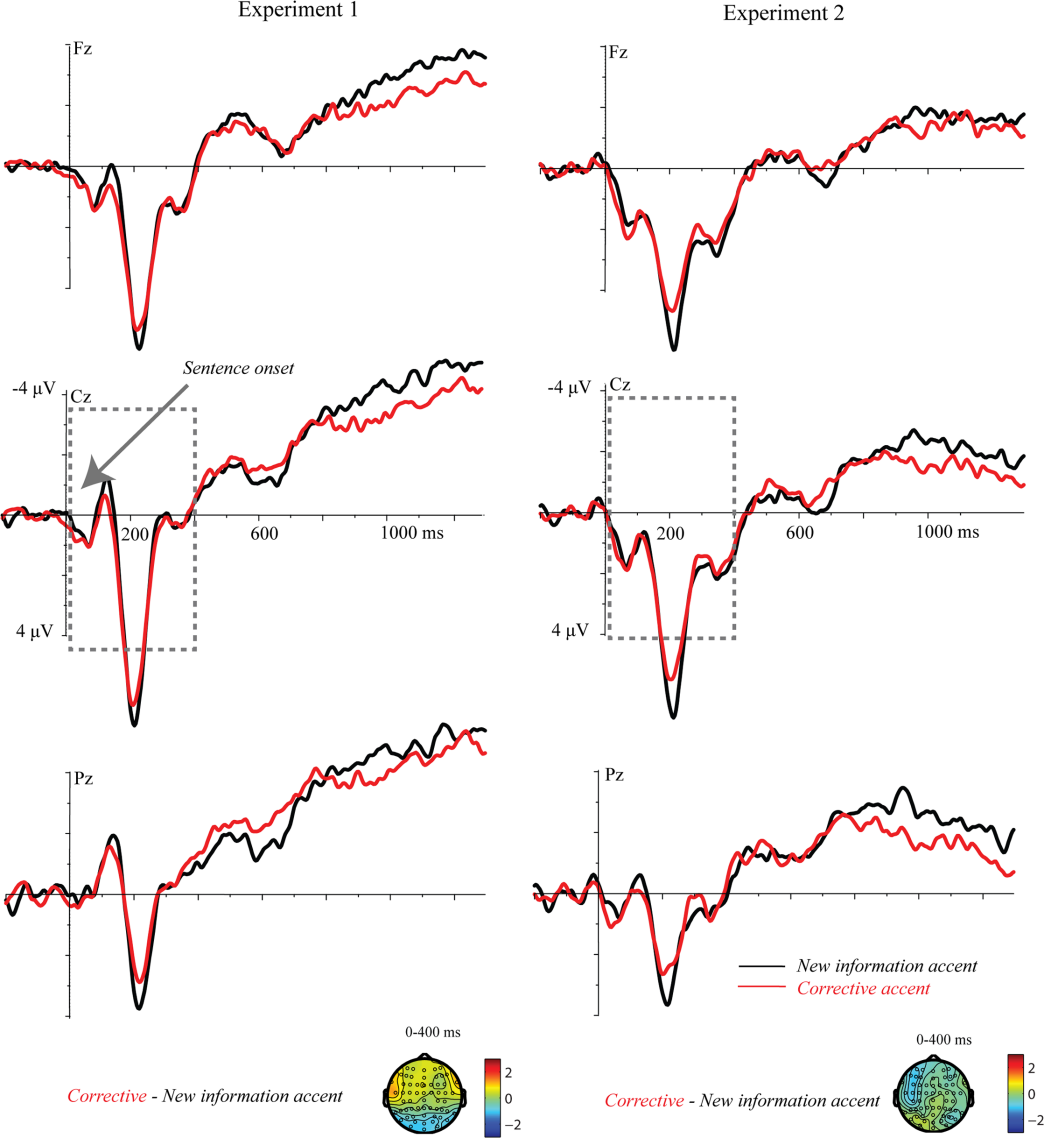
ERPs time-locked to sentence onset (experiment 1 and 2). ERPs display the effect of *Accent* and in sentences with new information accents (black lines) and sentences with corrective accents (red lines).

*0–400 ms*: The cluster-based random permutation tests did not reveal any difference in the processing of trials with corrective accents vs. trials with new information accents prior to the onset of the target word (CA vs. NA; no clusters). ERPs time-locked to sentence onset are displayed in Fig 4.

### Discussion of Experiment 1

Experiment 1 investigated how corrective and new information accents in Dutch modulate information processing in single sentences. The ERP results show that listeners process words carrying a corrective accent differently than words carrying a new information accent. This difference is reflected in a late positivity for corrective accents.

One possibility that we should examine before going to the predictions discussed in the introduction is that the differential neural processing of corrective accents seen here could reflect sensory processing, as corrective accents were acoustically more prominent than new information accents, rather than a distinct interpretation. Corrective accents had a higher pitch and a larger pitch slope, which may have caused a difference in ERPs. Previous research has shown that acoustic aspects of stimuli generally modulate fairly early ERP components such as the N1, the *Mismatch Negativity* (MMN, for a review, see [37]), and the P2, a positivity around 200 ms post stimulus onset [27], [34]. Friedrich et al. [34], for instance, showed that the P2 is related to processing words with falling pitch, unlike the rising pitch of corrective accents in the present study. As can be seen in Fig 4, there is a positivity peaking around 300 ms with a relatively frontal distribution, for both pitch accents. Thus, both sorts of accent appear to elicit an “accent positivity” which we have reported earlier for contrastive accents relative to unaccented words in context [7].

The extra processing cost of corrective accent was thus not primarily at the early sensory level. We also found late and long-lasting positive effects to corrective accents, starting around 500 ms after the onset of the accented word. Such late positivities may reflect recognition of the incongruity of the accent. This is in line with [23] who found that German listeners consider focus accents in single sentences as incongruent. In the ERP signal the words carrying corrective accents elicited a positivity over centro-posterior sites, relative to sentences with new information prosody. Under this interpretation, the neural mechanism underlying the late positivity for corrective accents in the present study might reflect processing costs due to an overspecified and less preferred prosodic prominence which the brain tries to resolve. This interpretation is in line with previous findings of late positive effects for incongruent accents in sentences in context [7], [35–36].

Alternatively the positive effect may represent re-interpretation processes when listeners consider corrective accents excessively prominent and *overspecified* in isolated sentences. Due to the lack of a preceding context, listeners cannot link nouns produced with corrective accents to an alternative noun, and hence the corrective accent is infelicitous as the correction cannot be resolved. One way the listener could try and resolve the infelicitous accentuation would be to compute an alternative to the noun to resolve the correction, as in *It was Tara*, *and not someone else (e*.*g*., *John)*, *who bought the flowers*, as some of the participants in Pretest 2 did. Behavioral and eye tracking evidence strongly support the hypothesis that online computation processes exist which fill in discourse models appropriately by means of similar inferences [8], [10], [13], [38]. Under this view, the ERPs for corrective accents suggest that listeners interpret these accents as carrying an additional meaning, which participants need to (re)construct. Unlike the case of new information accents, this process may require additional computation costs for corrective accents, which in turn may be reflected in the late positivity for corrective accents in our study. In experiment 2 we tested whether the effect seen in this experiment can best be interpreted as an effect of infelicity or as an effect of online construction of an unspecified alternative for correction.

## Experiment 2

The goal of experiment 2 was to further investigate the mechanism underlying the processing of corrective accents seen in experiment 1. If the effect reflects the infelicity of corrective accents in out-of-the blue sentences because they cannot be linked to an alternative in single sentences, it should disappear when sentences are embedded in supportive contexts. If the effect reflects additional computation costs related to the interpretation of the accented word as being a correction, it should persist even in context. Embedding sentences in contexts which do not support a felicitous interpretation allows us to examine whether correction context imposes requirements for a specific prosodic realization.

### Methods

#### Participants

Thirty-six right-handed Dutch native speakers (9 male, age 18–29, mean 21), none of whom participated in experiment 1, were paid for participation. Inclusion criteria were identical to experiment 1. Eight participants were excluded because their ERPs contained too many artifacts. The analysis was performed with data from the remaining twenty-eight participants.

#### Materials

As described in experiment 1, two Dutch native speakers recorded 120 short dialogues with corrective yes/no-questions and wh-questions asking for new information (see Table 1). After recording, questions and answers were cut out of the original dialogues and recombined. Corrective and new information questions were combined with answers with corrective or new information prosody. In total 480 dialogues (120 items x 2 accent types x 2 context types) were assigned to four experimental lists of 120 dialogues each (30 items per condition) using a Latin square to avoid repetition. Each dialogue was presented in all four conditions across lists, and no participant heard more than one version of each dialogue.

### EEG procedure

The experimental procedure is similar to experiment 1. Each trial started with a 100 ms delay, followed by a question that was presented auditorily via loudspeakers (average duration: wh-questions = 2000 ms, yes/no-questions = 2700 ms), followed by silence (500 ms), the answer (average duration of 2000 ms), and silence again (1500 ms). The semantic decision task and the EEG recording procedure are identical to experiment 1.

### ERP analysis

ERP analysis was performed on 98.3 percent valid data. As in experiment 1, ERPs were time-locked to target onset and to sentence onset, and ERPs were averaged across the same time windows: (i) time-locking to target onset: 100–300 ms, 300–500 ms, 500–1000 ms; (ii) time-locking to sentence onset: 0–400 ms. Statistical analyses were performed in Fieldtrip using cluster-based random permutation tests [32–33]. Importantly, cluster-based random permutation tests only allow for pair-wise comparisons of two conditions. The main effect of *Accent* was tested by comparing trials with corrective accent CA_CC+NC_ to trials with new information accent NA_CC+NC_, both collapsed over corrective context (CC) and new information context (NC). The main effect of *Context* was tested by comparing trials in corrective context CC_CA+FA_ to trials in new information context NC_CA+NA_, now collapsed over the accent conditions. To examine interactions between *Accent* and *Context*, we compared the difference waves of each two conditions (CC_CA-FA_ vs. NC_CA-NA_). If an interaction turned out to be significant, we split the data and performed simple effect analyses.

### Results of Experiment 2

#### Behavioral results

The behavioral task was identical to experiment 1. The average accuracy of 93% shows that participants paid attention to the stimuli.

#### ERP results time-locked to target onset

**Fig 5:**
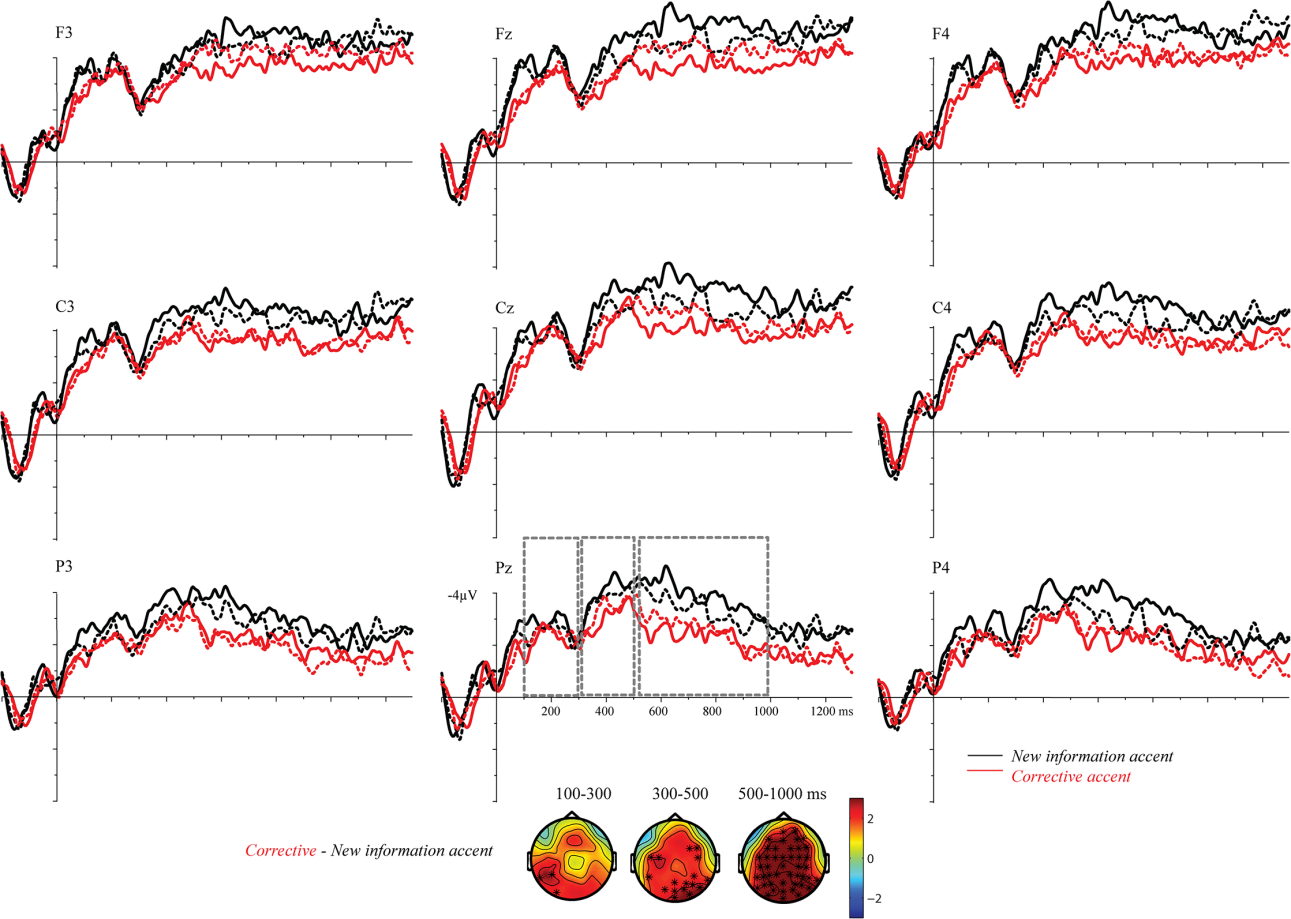
ERPs to sentences in context in experiment 2. ERPs are time-locked to the acoustic onset of the target *Tara* when produced with a corrective accent (red lines) or with a new information accent (black lines), either in a new information focus context (solid lines) or in a corrective focus context (dotted lines).

As can be seen in Fig 5, similar to experiment 1, the sentences containing corrective accent are in general more positive than those containing a new information accent, with the positivity beginning fairly early after onset of the target. Additional analysis of ERPs time-locked to sentence onset is presented in Fig 3.

*100–300 ms*: The cluster-based random permutation test revealed a difference between trials with a corrective accent vs. trials with a new information accent (CA_CC+NC_ vs. NA_CC+NC_; p<.05). The ERPs show that targets with corrective accent gave rise to a positivity. There was no main effect of *Context* (CC_CA+NA_ vs. NC_CA+NA_; no clusters) or an interaction of *Accent* and *Context* (CC_CA-NA_ vs. NC_CA-NA_; no clusters).

*300–500 ms*: We found that targets with corrective accents were processed differently than targets with new information accents (CA_CC+NC_ vs. NA_CC+NC_; p<.05), as evident in a positive effect to targets with corrective accents. There was no main effect of *Context* (CC_CA+NA_ vs. NC_CA+NA_; no clusters) or an interaction of *Accent* and *Context* (CC_CA-NA_ vs. NC_CA-NA_; no clusters).

*500–1000 ms*: The cluster-based random permutation tests showed that targets with corrective accents were processed differently than targets with new information accents (CA_CC+NC_ vs. NA_CC+NC_; p<.001). We did not find a main effect of *Context* (CC_CA+NA_ vs. NC_CA+NA_; no clusters) or an interaction of *Accent* and *Context* (CC_CA-NA_ vs. NC_CA-NA_; no clusters). As shown in Fig 5, corrective accents elicited a centro-parietal positivity, relative to new information accents.

#### ERPs time-locked to sentence onset

*0–400 ms*: The cluster-based random permutation test did not reveal any difference between trials with corrective vs. new information accents in this time window (CA_CC+NC_ vs. NA_CC+NC_; no clusters). There was also no main effect of *Context* (CC_CA+NA_ vs. NC_CA+NA_; no clusters) or an interaction of *Accent* and *Context* (CC_CA-NA_ vs. NC_CA-NA_; no clusters). ERPs time-locked to sentence onset are presented in Fig 3.

#### Summary of results

When sentences were embedded in a discourse context, listeners were already sensitive to differences in accentuation 100 ms after the onset of the target word. Corrective accents triggered a positivity relative to new information accents.

### Discussion of Experiment 2

In this experiment we investigated whether discourse context modifies the processing of words produced with a corrective accent relative to a new information pitch accent. We found a positivity for corrective accents, similar to the positivity for corrective accents in the out-of-the-blue sentences in experiment 1. The context had a clear effect in that the positivity for corrective prosody started earlier in context than in isolated sentences, before 300 msec after the onset of the accented word. The positivity was not significantly modulated by the type of preceding context, however.

The lack of an interaction between prosody and discourse context is surprising under an interpretation where the corrective accent carries the specific meaning of ‘correction’, which should make these accents rather unacceptable in new information contexts, where there is no alternative to correct, in comparison to the corrective contexts, where an alternative is available. In contrast to that expectation, our ERP findings show that words produced with corrective accents are processed similarly regardless of the corrective or new information context preceding it. What seems to play a role is the *presence*, rather than the type of discourse context, as in context the difference between new information and corrective prosody becomes apparent almost immediately after the onset of the accented word. Our findings appear, in principle, compatible with both hypotheses we started out with: corrective accents have a corrective meaning, versus corrective accents are merely more emphatic and are interpreted depending on the context.

According to the first hypothesis, the corrective accent is interpreted as indicating correction and initiates a process of identifying and correcting an element in the context. In the corrective contexts, the positivity would reflect this process. In wh-contexts, no contextual alternatives are present and listeners need to hypothetically infer them, as Pretest 2 and previous experiments on English “only” have shown [39]. The positivity then reflects the same process except that the alternative is never fully specified and thus cannot be replaced.

Under the second hypothesis, a corrective accent could have a number of different functions or meanings, simply indicating that something special is going on, due to their highly emphatic profile. From a pragmatic perspective, the Gricean Maxim of Quantity (*Be as informative as required*, *and not more informative*) [40] would lead listeners to attempt to interpret targets produced with this emphatic accent as providing relevant information. In contexts where an alternative is present, correction can be inferred. In the wh-question context, this is not possible, and inferencing could take another form. For instance, the reader might suspect the emphatic accent was in place to indicate that the action portrayed in the target sentence was very much out of character for the specific individual (e.g., for Tara in “I think TARA bought the flowers”). The inferencing process is then assumed to produce the late positivity.

## General Discussion

In the introduction, we discussed the status of corrective accents in Dutch and distinguished two viewpoints. First, pitch accents may carry distinct meanings or functionality, such as introducing new information in the discourse (new information accents with falling pitch) or implying correction (corrective accents with rising pitch) and hence corrective accents are only appropriate in a specific context where alternatives are present. Second, corrective pitch accents could belong to a continuum where emphasis is enhanced acoustically but does not distinguish between a corrective vs. new information meaning per se and hence would be appropriate in many contexts. We tested these hypotheses in two ERP experiments where words carrying different accents were either produced in single sentences or in sentences which served as a reply to corrective or new information questions. Our data demonstrate that processing corrective accents is distinct from the processing of new information accents. Moreover, corrective accents are processed similarly regardless of whether a clear alternative is present in the discourse context, though context generally seems to speed up the recognition of different types of accents. In each case, corrective accents are associated with a late positivity, as compared to the new information accents.

This positivity brings to mind several studies that have shown distinct late positive effects when listeners process incongruent linguistic information [41–43]; for review see [44–46] or when listeners’ integration of linguistic information is more effortful, in the lack of any obvious incongruity [47–48]. In particular there are a number of previous studies on prosody that have also reported late positive ERP components when listeners have difficulties integrating prosody in context. In our previous study we argued that such positivities may belong to the family of the P600 component, suggesting that a disagreement between prosody and information structure increases the costs for integrating information into a coherent discourse representation [7]. A recent fMRI study provides further evidence that the disagreement between prosody and information structure increases unification costs [49]. Late positivities have also been reported for German pitch accents when they do not match the degree of activation of the referent in the context, e.g. strong prosodic prominence on a recently introduced and thus active noun [35]. Hence, the neural mechanism underlying the late positivity for corrective accent could correspond to processes of integrating words with less appropriate accentuation into the discourse context.

The positivity for corrective accents occurs even when an alternative is available for correction in the context. This suggests that the processing cost reflected in the late positivity does not reflect incongruity per se. Rather it suggests that the additional processing efforts for corrective accents might reflect the effort of inferring its relation to context, present or not. When there is nothing to correct, as in single sentences and in new information contexts, corrective accents suggest that there is nevertheless some reason to pay special attention. Following the Gricean maxim of *Relevance* listeners then try to create inferences to make sense of the signal, which gives rise to a late positivity [38], [40].

This account is in principle compatible with both views of corrective accent discussed in the introduction. Under the view that the more marked accent does not have a specific meaning, but is interpreted in context, it may be the case that the accent evokes a general process of inferencing in which listeners attempt to construct an appropriate meaning for the extra accentuation. The most likely interpretation when an alternative is present would be that the speaker intends correction. However, in a null context or a context with no alternative, other possibilities may be considered, such as the general incongruity of the factual answer (i.e., *Who would have expected* ***Petra*** *to buy the flowers*?) or the semantic anomaly of the accented information with respect to world knowledge (i.e., *the BLUE banana*) [50].

The results can also be explained by the hypothesis that corrective accent has a specific meaning. As pointed out in the introduction, listeners could engage additional efforts to perform correction as a result of the more emphatic corrective accent. In this view corrective accent does have a meaning, namely “corrective”, and it instigates corrective processing. Performing a correction is a twofold process: listeners identify that a correction is being made on the basis of the prosodic contour, and then an alternative must be identified in the discourse that needs to be replaced. With an alternative in the preceding context, listeners link the accented word in the answer to that alternative in the question, whereas without an alternative in isolated sentences or new information question contexts, listeners may need to construct an unspecified or plausible alternative referent first which the accented name implicitly refers to.

The results of pretest 2 provide some behavioral evidence for the existence of such a process. When required to explicitly imagine a preceding context to sentences with corrective and non-corrective accents, some listeners construct a discourse context with an alternative referent (e.g., *John*) upon perceiving a corrective accent on *Tara*. Our data are thus compatible with the prediction that a distinct processing mechanism is initiated by corrective accents.

This interpretation is in line with previous behavioral and eye tracking findings about the role of contrastive accents in other Germanic languages, suggesting that prosody can initiate a process of constructing a discourse representation with an appropriate meaning [8], [10], [13], [51]. According to these studies contrastively accented words initiate the expectation of a contrast which results in a higher number of eye movements directed to contrastive elements in the discourse. Furthermore, previous studies have demonstrated that listeners remember contrastively accented words better than words with new information accents [52–53]. The neural signature underlying this process has been unexplored so far, and the present data suggests that it might be linked to the family of late positivities as proposed by [44], who argue that these positivities reflect processing towards arriving at a coherent mental representation of what is communicated.

Lastly, an interpretation where the positivity for corrective accents reflects the processing of a mere acoustic emphasis without the implication of correction cannot be fully ruled out in the present design. As suggested in the discussion of experiment 2, the late onset of the positive effect for corrective prosody, particularly in single sentences, speaks against it being due to pure acoustic emphasis, which usually modifies early stages of sensory processing. The length of the effect in context also speaks against this interpretation. We additionally examined whether pitch differences prior to the target word can account for the observed positive effect of prosody and time-locked the ERP signal to the sentence onset. The sentence onset analysis is sensitive to sensory effects which are elicited by prosody, but relatively insensitive to effects time-locked to the onset of the target noun, due to jitter associated with the length of the individual items and the more systematic differences in onset due to length differences in the two prosodic patterns. The results of the sentence onset analysis for both experiments suggest that prior to the target word sentences with corrective and new information accents do not show a statistically reliable difference.

In sum, in the present study we focus on corrective accents in comparison with the processing of new information accents. The results suggest that corrective accents induce an attempt to construct an appropriate context or make an appropriate adjustment to context. However, we were not able to determine whether this was due to the emphasis alone, leading to a general inferencing process, or a construction of a correction. A potential extension of this study would be to directly compare contrastive and corrective prosody. In the introduction we discussed the potential three-way distinction between new information, contrastive and corrective focus [4]. Some evidence about the realization of contrastive prosody can be found in our previous ERP study [7] where we recorded stimuli in contrastive contexts (*Did they give a bonus or a fine to the player*?*—They gave a bonus to the player*). In this experiment contrastive accents on “bonus” were produced with falling pitch and had the shape of a default focus accent (H*L) in Dutch, although the fall was more extended. Hence we have some reason to assume that corrective prosody is distinct and special, in that it is more distinguishable and prominent than contrastive prosody in Dutch. Further experimentation would provide clarification of this issue.

## Supporting Information

S1 Table Stimulus Materials. The table contains the experimental materials used in both experiments. In experiment 1 we used only the answer sentence in the two accent conditions (corrective accent vs. new information accent). In experiment 2 we used both the question and the corresponding answer.
(XLSX)
